# Prevalence of obstructive sleep apnoea in sleep consultations in Burkina Faso: Implications for monitoring

**DOI:** 10.7196/AJTCCM.2020.v26i3.042

**Published:** 2020-10-13

**Authors:** A R Ouédraogo, A Tiendrebeogo, K Boncoungou, E Birba, G A Ouédraogo, M M Assao Neino, G Bougma, G Ouédraogo, G Badoum, M Ouédraogo

**Affiliations:** 1 Department of Pulmonology, University Hospital Yalgado Ouedraogo, Ouagadougou, Burkina Faso; 2 Department of Pulmonology, University Hospital Souro Sanou, Bobo Dioulasso, Burkina Faso; 3 Department of Pulmonology, Regional Hospital of Ouahigouya, Ouahigouya, Burkina Faso; 4 Department of Pulmonology, National Hospital Lamordé, Niamey, Niger

**Keywords:** Obstructive sleep apnea syndrome, Respiratory polygraphy, CPAP, Burkina Faso

## Abstract

**Background:**

Obstructive sleep apnoea syndrome (OSAS) is the most common respiratory disorder related to sleep. Its prevalence in developed countries varies from 3% to 28%. In several African countries, including Burkina Faso, this syndrome is still under-diagnosed and goes largely untreated. It is necessary to conduct studies in different contexts to determine the characteristics and develop the strategies for management of OSAS.

**Objectives:**

To determine the prevalence of OSAS in Burkina Faso.

**Methods:**

This prospective study recruited 106 patients coming for consultation for sleep disorders at the Yalgado Ouedraogo University Hospital Center, who responded to a self-questionnaire and were diagnosed by respiratory polygraphy.

**Results:**

A total of 77 patients (72.6%) had OSAS. The male to female ratio was 1.4:1 and the mean (standard deviation) age was 47.8 (12.8) years. The majority of the patients (53.8%) were obese. The main reason for consultation was snoring (84%), followed by hypopnea-apnoea reported (59.4%) and daytime sleepiness (45.3%). The most common comorbidity factor was hypertension (50%), followed by decreased libido (16%) and diabetes (13.2%). A continuous positive-pressure (CPAP) machine was prescribed to 51.25% of the patients, but only 22% were able to acquire it.

**Conclusion:**

The monitoring of OSAS is relatively new in Burkina Faso. This study showed the profile of patients with OSAS and difficulties in accessing continuous positive airway pressure (CPAP) devices for treatment.

## Background

Obstructive sleep apnoea syndrome (OSAS) is the most common sleep-related breathing disorder. It is characterised by a cessation or a decrease of respiratory air flow.^[1]^ OSAS affects nearly 5% of the global adult population and is responsible for numerous cardiovascular complications such as hypertension, coronary artery disease, stroke and heart failure.^[2]^ The disease is marked by a deterioration in quality of life.^[2,3]^ Therefore, OSAS represents a significant public health problem.

Sixty percent of OSAS patients remain undiagnosed globally.^[4]^ The risk factors for OSAS are multiple and include being overweight, age, male gender, certain endocrine pathologies and morphological predispositions to upper airway obstruction.^[5]^ In several African countries, including Burkina Faso, OSAS is still under-diagnosed owing to the expensive equipment required to record sleep disordered breathing. The diagnosis of OSAS is based on a night recording of breathing using either polysomnography or respiration polygraphy. Accessibility to treatment is also a major challenge. Because of the cardiovascular complications and the large population that goes undiagnosed, it is important to investigate the prevalence of this condition in Burkina Faso.

## Methods

### Framework and study population

This study took place in Ouagadougou, the capital city of Burkina Faso. The population of Burkina Faso was estimated at 19 034 397 inhabitants in 2016, with 2 637 303 inhabitants living in Ouagadougou.^[7]^ It is a landlocked country located in West Africa.

The study was carried out in the Pulmonology Department of Ouagadougou University Hospital Yalgado Ouedraogo (CHU-YO), which has the only public unit for the management of sleep-related respiratory diseases in Burkina Faso. The consultations were conducted twice a week by two pulmonologists trained in the management of sleep-related breathing disorders. The unit had only one CIDELEC 102L respiratory polygraph machine (CIDELEC, France). The cost of testing was USD54.07. Patients who were unable to pay these fees were exempted.

Burkina Faso does not have a supplier for the marketing and maintenance of continuous positive airway pressure (CPAP) devices. CPAP devices are ordered from abroad by patients. Titrations and follow-ups are provided by the unit’s doctors. In the private sector, only one medical clinic was able to diagnose OSAS using respiratory polygraphy.

### Population and type of study

This was a prospective study conducted from 1 April 2016 to 31 March 2017 in the Pulmonology Department of CHU-YO. It involved patients with functional OSAS symptoms who were referred by other practitioners or who presented on their own for sleep consultation.

### Data collection

An anonymous survey record collected sociodemographic characteristics, past medical histories, clinical data, polygraphic and therapeutic data. The Epworth scale was used to evaluate daytime sleepiness and the Pichôt scale for fatigue. A CIDELEC CID 102L respiratory polygraph (level III (Cidelec, France)) was used for the recordings. The polygraph included a continuous oximetry sensor, tracheal sound sensor, suprasternal pressure sensor, nasal flow sensor (nasal canula), and sensors for thoracic and abdominal movements.

The questionnaire was administered during the consultation as an interview and explained in the local language for patients who did not speak French. Details of the history of the disease, the past medical history, and a physical examination with measurement of the anthropometric parameters (weight, height, neck and abdominal circumference) were recorded. The patients were then referred for an ear, nose and throat (ENT) consultation for a possible local cause. On the night of the polygraphic recording, an intern (7th year of medicine) measured the pulse and the blood pressure. The reading of the results was carried out by a pulmonologist trained on sleep-related breathing pathologies.

OSAS is defined by the presence of criteria A or B and C:

A. Excessive daytime sleepiness not explained by other factors;
  B. At least two of the following criteria not explained by other factors: severe daily snoring, sensations of choking or suffocation during sleep, unrepairing sleep, daytime fatigue, difficulty concentrating, nocturia (more than one urination per night);
  C. Polysomnographic or polygraphic criteria: apnoeas + hypopnoeas ≥5 per hour of sleep (Apnoea-Hypopnoea Index (AHI) ≥5).^[11,13]^

We define a casual snorer as a person who snores less than three nights a week and a usual snorer as a person who snores more than three nights a week.^[12]^ Other operational definitions are summarised in (Table. 1).

Depending on the severity, clinical impact and results of the investigations, the appropriate treatment was proposed according to the 2010 French recommendations for clinical practice.^[13]^

CPAP was recommended as initial therapy for patients with severe OSAS even in the absence of symptoms and moderate OSAS with excessive daytime sleepiness and/or cardiovascular or respiratory comorbidities.

A mandibular advancement device (MAD) was recommended for patients with mild to moderate OSAS in the absence of symptoms and comorbidities, or for patients who declined or failed to adhere to positive airway pressure therapy or who had a preference for this treatment.

Weight loss and exercise were recommended to all patients with OSAS who were overweight or obese. Positional treatment, which consisted of avoiding the supine position during sleep, has been recommended for mild to moderate positional OSAS in the absence of significant obesity.

### Statistical analysis

The data were analysed using the Epi Info 7.2 statistics software (Centres for Disease Control and Prevention, USA). Pearson’s Gross χ2 test or Fischer’s exact test were used to compare categorical variables. Mean values were presented with the standard deviation as the dispersion index. Pearson’s linear correlation coefficient (r) was used to measure correlation between quantitative variables. Associations between variables were considered statistically significant at the probability threshold of p<0.05.

### Ethical considerations

The study was carried out with respect for the anonymity and confidentiality of the information collected. It was carried out in accordance with bioethical laws and with good clinical practice. We obtained informed consent from all participants before enrolment.

## Results

### Sociodemographic and clinical data

During the study period, 1 784 patients were seen in the Pulmonology Department of CHU-YO. Among these, 5.9% (n=106) came for sleep consultation. The sociodemographic and clinical data for all the patients are summarised in (Table. 2).

The male to female ratio was 1.4:1. The average (SD) age of patients was 47.8 (12.8) years with extremes of 18 and 79 years. There were 78.3% (n=83) patients who were employed. The percentage of married and college-educated patients was 78.3% (n=83) and 51.9% (n=55), respectively. Snoring was the main reason for consultation and was reported by 84% (n=89) of the patients, followed by the feeling of choking during sleep in 59.4% (n=63) of patients. Hypertension was the main comorbidity in 50% (n=53) of the patients. More than a third of the patients 36.8% (n=39) consumed alcohol, while 18.9% (n=20) were smokers. Obese patients accounted for 53.8% (n=57) of the study population. The mean (SD) body mass index (BMI) was 31.7 kg/m² (6.9) with extremes of 19 and 50 kg/m². Neck and abdominal circumferences were excessive in 37.7% (n=40) and 70.8% (n=75) of the patients, respectively. More than a third of the patients (36.8%; n=39) felt that the duration of their sleep was not enough. Excessive fatigue was found in 41.5% (n=44) of patients using the Pichôt scale. A quarter of the patients (25.5%; n=27) had a sleep deficit and 21.7% (n=23) had scores of excessive daytime sleepiness as determined using the Epworth scale. There was a strong positive correlation between fatigue and drowsiness (r=0.64; p<0.001). The ENT examination identified cases of sinusitis (9.4%; n=10), macroglossia (6.6%; n=7) and nasolabial polyposis (2.8%; n=3).

### Respiratory polygraphy and treatment

**Fig. 1:**
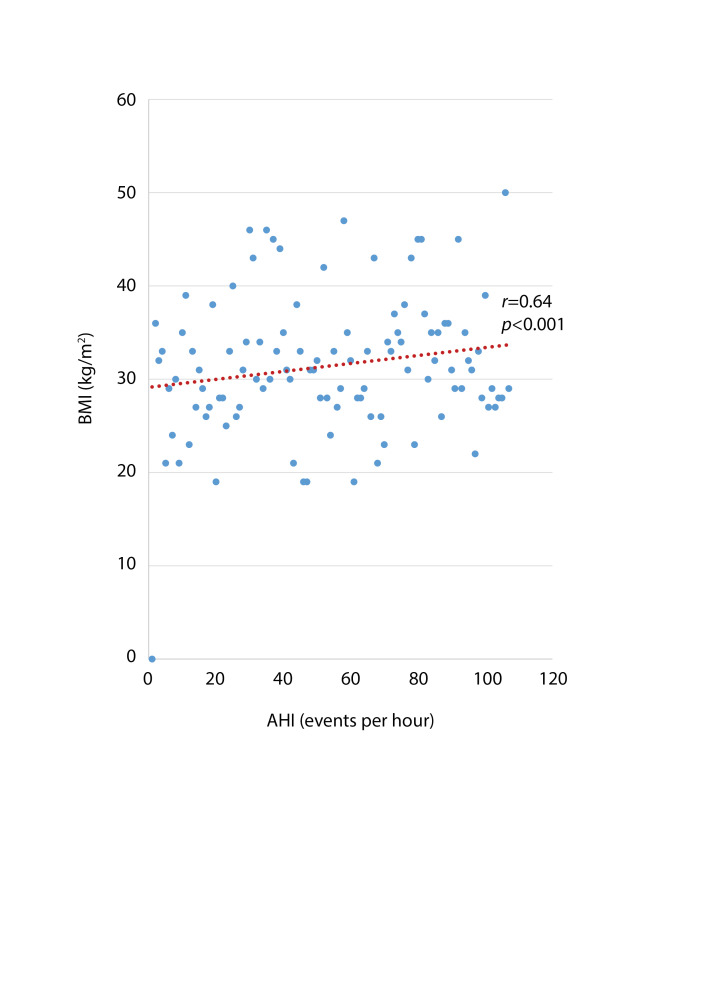
Correlation between AHI and BMI. (AHI = apnoea-hypopnoea index; BMI = body mass index.)

**Fig. 2:**
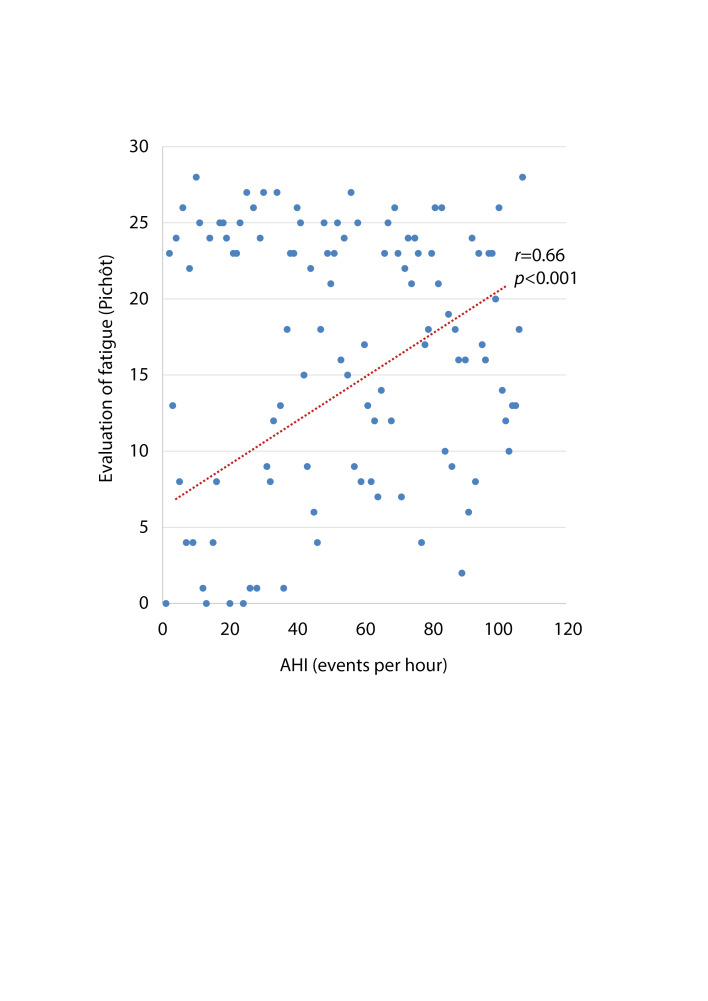
Correlation between AHI and fatigue assessment on the Pichôt scale. (AHI = apnoea-hypopnoea index.)

**Fig. 3:**
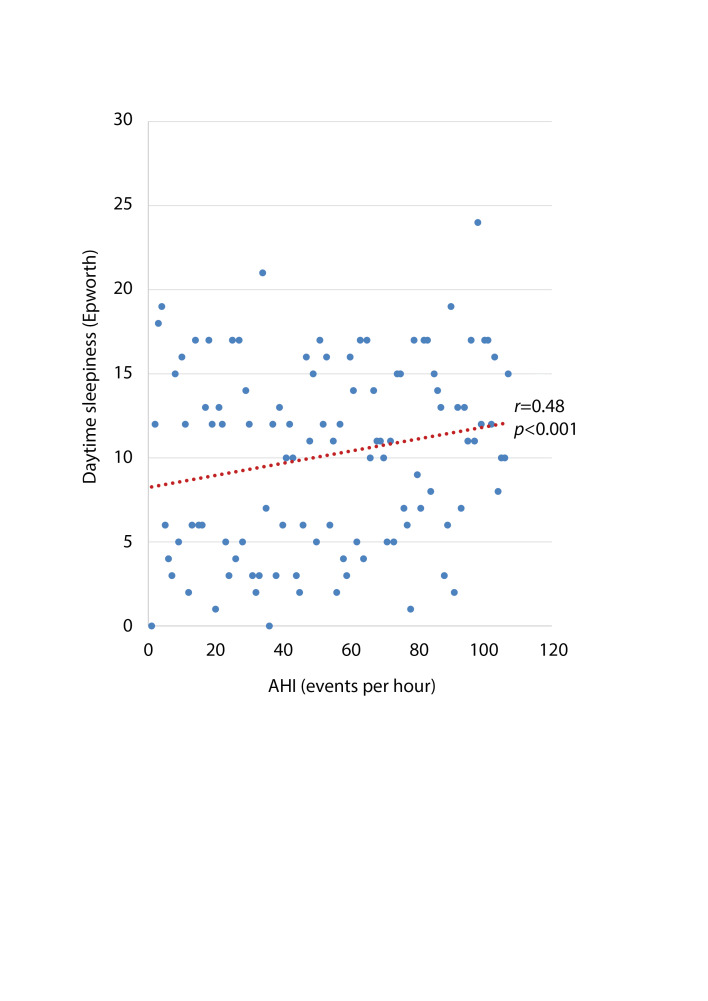
Correlation between AHI and daytime sleepiness on the Epworth scale. (AHI = apnoea-hypopnoea index.)

Respiratory polygraphy was performed in all patients and the results are summarised in (Table. 3). The average (SD) duration of the recording was 7 (0.7) hours. OSAS was confirmed in 72.6% (n=77) of the patients and was classified as severe in 28.3% (n=30) of the patients. The predominant position of apnoea occurrence was dorsal decubitus (98.7%). Mean (SD) SpO_2_ at wakefulness was 96% (2.2), with extremes of 83% and 98%; at night it was 94.1% (3.6), with extremes of 76% and 98%. There was a strong positive correlation between AHI and nocturnal SpO_2_ r=0.63; p<0.001). The oxygen desaturation index (ODI) was ≥20 episodes per hour in 49.1% (n=52) patients. The snoring index (SI) revealed that 30.2% (n=32) of the patients were severe snorers. The factors associated with OSAS are summarised in (Table. 4). There was a positive correlation between AHI and neck circumference (NC) (r=0.45; p<0.001) and abdominal circumference (AC) (r=0.48; p<0.001). There was also a correlation between AHI and BMI (Fig. 1), Pichôt fatigue scale (Fig. 2) and Epworth daytime sleepiness scale (Fig. 3).

All patients with OSAS were advised to change their lifestyle and diet. Postural treatment (tennis ball in a vest to stop the patient lying on their back) was offered to 11.7% (n=9/77) of the patients. A MAD device was indicated in 35% (n=27/77) of the patients; however, no patient was able to purchase the machine. CPAP treatment was prescribed to 53.2% (n=41/77) of the patients. However, only 21.9% (n=9/41) of these patients were able to acquire the CPAP device. The lack of financial means for the acquisition of the device was mentioned by 78.1% (n=32/41) of the patients. A total of 88.7% (n=68) of patients with OSAS were lost to follow-up.

## Discussion

Our study concerned patients referred for a sleep consultation with functional symptoms suggestive of OSAS. The monitoring of OSAS is relatively new in Burkina Faso. This was the first study in the country to describe the incidence of OSAS. We found that the prevalence of OSAS was 72.6% in our study. Studies in Vietnam^[14]^ and Morocco^[15]^ reported a prevalence of 87.1% and 56.7%, respectively.

In our study, the majority of patients were men (58.5%), who were 2.6 times more likely to have OSAS than women (odds ratio (OR) 2.6; 95% confidence interval (CI) 1.1 - 6.3; p=0.04). The literature states that the prevalence of OSAS is higher in men than in women (sex ratio of 3:1) due to morphological differences and hormonal factors, especially in the first five decades.^[16,17]^

Beyond the age of 50, the prevalence of OSAS in women increases, suggesting that menopause may be a risk factor for OSAS and hormone replacement therapy may be protective.^[16,17]^ The average (SD) age of our patients was 47.8 (12.8) years with extremes of 18 and 79 years. The prevalence of OSAS increased almost linearly in adults up to age 65, independently of other risk factors.^[18]^ More than half (54.7%) of the patients were obese. There was a correlation between the AHI and NC (r=0.45; p<0.001) and AC (r=0.48; p<0.001).

Excessive AC was associated with onset of OSAS (OR 2.6; 95% CI 1.1 - 6.5; p=0.03). Obesity is classically known to be a risk factor for OSAS because it can cause narrowing of the airways due to excess fatty tissue around the neck. Several epidemiological studies have confirmed an increase in the prevalence of OSAS in overweight individuals.^[5,18]^

Snoring, often noted by a spouse, was the most common reason for consultation (84%) in the present study. Sixty percent of men and 40% of middle-aged women (40 - 60 years) are habitual snorers.^[19]^ Snoring is almost constant in OSAS.^[19,20]^ However, snoring is not synonymous with OSAS.

More than half of our patients (54.3%) presented with excessive fatigue and 21.7% with excessive daytime sleepiness. These signs are thought to be responsible for a concentration disorder, memory problems and a decline in intellectual performance.^[18,21]^ This could have repercussions on the professional performance of the 78.3% of patients in our study who were employed. The feeling of choking felt by the patients (59.4%) and/or the occurrence of respiratory pauses (21.7%) was due to obstruction of the upper airways.

Half of our patients had hypertension. OSAS is a major cause of hypertension.^[22]^ A proportion of 30% to 40% of patients with hypertension would have OSAS and 50% of patients with OSAS would be hypertensive.^[22]^ The results of many clinical studies strongly suggest that OSAS is an independent risk factor for cardiovascular diseases such as hypertension, coronary artery disease, stroke and heart failure.^[22]^ Several mechanisms have been suggested to link OSAS and vascular diseases, including increases in sympathetic activation, oxidative stress, inflammation, endothelial dysfunction, coagulation and metabolic dysregulation.^[22]^

Asthma was present in 17% of our patients. Asthmatics have an increased risk of sleep apnoea and apnoea can worsen asthma by increasing inflammation.^[23]^ Recent data suggested that OSAS is an independent risk factor for asthma exacerbations.^[23]^ Neuromechanical reflex bronchoconstriction, gastroesophageal reflux, inflammation (local and systemic), and the indirect effect on dyspnoea of OSAS-induced cardiac dysfunction have been suggested as mechanisms that lead to worsening asthma in patients with concomitant OSAS. In patients whose asthma is not controlled by drug therapy, the presence of OSAS should be investigated and properly managed.^[23,24]^

Diabetes was found in 13.2% of our patients. OSAS is a risk factor for diabetes. A study by Shaw^[25]^ found that 40% of people with OSAS have diabetes and that the prevalence of OSAS can reach 23% in people with diabetes.

The diagnosis of OSAS was established using respiratory polygraphy (level 3 portable respiratory polygraphy with at least 4 signals) in 72.6% of our patients. Polysomnography in a sleep laboratory is the reference examination for the diagnosis of OSAS.^[13]^ However, it is expensive and not available in Burkina Faso. The diagnostic performance of ventilatory polygraphy was compared with polysomnography. The results of these studies showed that ventilatory polygraphy in a patient with a presumptive clinical diagnosis confirms the diagnosis of OSAS with good specificity.^[13]^ Level 3 portable devices showed good diagnostic performance compared with level 1 sleep tests in adult patients with a high pre-test probability of moderate to severe OSAS and no unstable comorbidities. For patients suspected of having other types of sleep-disordered breathing or sleep disorders not related to breathing, level 1 testing remains the reference standard.^[26]^

Depending on the severity and clinical impact, adequate treatment has been proposed to our patients on the basis of the French 2010 recommendations for clinical practice.^[13]^ All patients with OSAS associated with obesity or who were overweight have benefited from lifestyle and dietary measures to reduce their weight. These measures included nutritional counselling as part of a comprehensive nutritional management. The placement of a MAD device was indicated in 35% of the patients, but all patients reported they could not obtain the MAD due to its unavailability and the very limited number of trained stomatologists/dentists. CPAP treatment of OSAS was prescribed to 53.2% of the patients, but only 21.9% of these patients were able to acquire the CPAP device. Since the first demonstration of the effectiveness of ventilation in the treatment of OSAS in1981, numerous studies have been published confirming its effectiveness on the regression of nocturnal respiratory disorders and the clinical symptoms associated with this pathology.^[13]^ Burkina Faso does not have a service provider for the marketing and maintenance of CPAP devices. CPAP devices are ordered from abroad by patients; however, many patients cannot afford the devices. This situation makes it difficult to care for patients with OSAS in a country with competing health priorities. Moreover, it is difficult to retain patients in the healthcare chain, as demonstrated by a loss of 88.7% of our patients during follow-ups.

## Conclusion

The monitoring of OSAS is relatively new in Burkina Faso. The present study showed some of the characteristics of patients with OSAS. Importantly, we showed that OSAS had a prevalence of 72.6%, in patients with sleep disorders. The majority of patients suffering from OSAS could not access therapeutic devices due to their high costs. Therefore, there is an urgent need to set up a multidisciplinary working group to train practitioners and to develop adequate means for better monitoring of this disease.

## Figures and Tables

**Table 1:** Operational definitions

EDSS^[8]^	Pichôt Fatigue Scale^[9]^		AC and NC^[10]^		Level of severity of the OSAS as defined by the AHI^[11]^	Quantification of snoring as classified in^[12]^
Score <11: normal vigilance	- Score ≤22: normal - Score >22: excessive fatigue		Normal if: Men: AC<94cm and NC<43cm		Mild: between 5 and 15 events per hour	Non-snorer: person whose SI is <30/h
Score between 11 and 15: sleep deficit	-		Women: AC<80cm and NC<41cm		Moderate: between 15 and 30 events per hour	Moderate snorer: SI between 30 and 100/h
Score ≥16: signs of excessive daytime sleepiness	-		-		Severe: 30 and more events per hour	Average snorer: SI between 100 and 300/h
-	-		-		-	Severe snorer: SI>300/h

EDSS = Epworth Daytime Sleepiness Scale

SI = snoring index

AC = abdominal circumference

NC = neck circumference

AHI = apnoea-hypopnoea index

**Table 2:** Sociodemographic and clinical characteristics (*N*=106)

Variables	Frequency, *n *(%)	
Gender	
Male	62 (58.5)
Female	44 (41.5)
Age (years)	
<30	8 (7.5)
30 - 39	21 (20)
40 - 49	35 (33)
50 - 59	25 (23.5)
≥60	17 (16)
Marital Status	
Married	83 (78.3)
Other	23 (21.7)
Professional status	
Active	83 (78.3)
Inactive	23 (21.7)
BMI (kg/m^2)^	
18.5 - 25	15 (14.2)
25 - 30	33 (31.1)
>30	58 (54.7)
NC	
Normal	66 (62.3)
Excessive	40 (37.7)
AC	
Normal	31 (29.2)
Excessive	75 (70.8)
Past medical history/comorbidities	
Smoking	20 (18.9)
Alcohol consumption	39 (36.8)
HTN	53 (50)
Decreased libido/erectile dysfunction	20 (18.9)
Asthma	18 (17.0)
Depression	18 (17.0)
Diabetes	14 (13.2)
Cardiopathies	8 (7.5)
COPD	1 (0.9)
HIV	1 (0.9)
Chief complaint	
Snoring	89 (84.0)
Feeling of choking/suffocation during sleep	63 (59.4)
Morning fatigue	59 (55.7)
Nocturia	56 (52.8)
Daytime sleepiness	48 (45.3)
Morning headaches	35 (33.01)
Respiratory pauses	23 (21.7)

**Table 3:** Respiratory polygraphy data (*N*=106)^*^

	Frequency, *n *(%)	
AHI (/h)	
<5	26 (24.5)
5 - 15	30 (28.3)
15 - 30	20 (18.9)
>30	30 (28.3)
Mean nocturnal SpO_2_ (%)	
<95%	67 (63.2)
≥95%	39 (36.8)
SpO_2_ on waking up (%)	
<95%	26 (24.5)
≥95%	80 (75.5)
ODI	
<10	29 (27.3)
10 - 20	25 (23.6)
≥20	52 (49.1)
SI	
Non-snorer	34 (32.1)
Moderate snorer	18 (17)
Average snorer	22 (20.7)
Severe snorer	32 (30.2)
Type of sleep apnoea syndrome (*n*=80)	
Obstructive	77/80 (96.3)
Central	3/80 (3.7)
Predominant position of apnoea occurrence (*n*=80)	
Supine position	79 (98.7)
Left lateral decubitus	47 (58.7)
Right lateral decubitus	41 (51.2)
Ventral decubitus	18 (22.5)

AHI = apnoea-hypopnoea index

ODI = oxygen desaturation index

SI = snoring index

^*^N=106 unless otherwise specified

**Table 4:** Factors associated with OSAS

Variable	OSAS, *n* (%)	OR (95% CI)	*p*-value
Gender			
Female	27 (61.4)	2.6 (1.1 - 6.3)	0.04
Male	50 (80.6)		
Age (years)			
<48	39 (69.6)	0.7 (0.3 - 1.7)	0.4
≥48	38 (76)		
NC			
Normal	46 (69.7)	1.5 (0.6 - 3.7)	0.38
Excessive	31 (77.5)		
AC			
Normal	18 (58.1)	2.6 (1.1 - 6.5)	0.03
Excessive	59 (78.7)		
History of hypertension			
Yes	41 (77.4)	0.6 (0.3 - 1.5)	0.27
No	36 (67.9)		
Smoking			
Yes	17 (85)	0.4 (0.1 - 1.5)	0.16
No	60 (69.8)		
Alcohol consumption			
Yes	31 (79.5)	0.5 (0.2 - 1.4)	0.23
No	46 (68.7)		
Sufficient sleep			
Yes	49 (73.1)	0.9 (0.3 - 2.2)	0.88
No	28 (71.8)		
Pichôt scale			
≤22	37 (59.7)	6.7 (2.1 - 21.2)	<0.001
>22	40 (90.9)		

OSAS = obstructive sleep apnoea syndrome

OR = odds ratio

CI = confidence interval

NC = neck circumference

AC = abdominal circumference
